# The Influence of Mucinous Histology on the Prognosis of Stage II and III Colorectal Cancers

**DOI:** 10.3390/medicina61030456

**Published:** 2025-03-06

**Authors:** İsa Caner Aydin, Mehmet Torun, Mehmet Reşit Sönmez, Serkan Ademoğlu, Ahmet Orhan Sunar, Orhan Uzun, Selçuk Gülmez, Erdal Polat, Mustafa Duman

**Affiliations:** 1 Department of Gastroenterologic Surgery, Ministry of Health Zonguldak Ataturk State Hospital, 67030 Zonguldak, Türkiye; 2 Department of Gastroenterologic Surgery, Ministry of Health Erzurum City Hospital, 25240 Erzurum, Türkiye; 3 Department of Gastroenterologic Surgery, Dumlupinar University Kütahya Evliya Çelebi Training and Research Hospital, 43040 Merkez, Türkiye; 4 Department of Gastroenterologic Surgery, Ministry of Health Gaziantep City Hospital, 25240 Erzurum, Türkiye; 5 Department of Gastroenterologic Surgery, University of Health Koşuyolu High Specialization Education and Research Hospital, 34668 Istanbul, Türkiye

**Keywords:** colorectal cancer, mucinous component, mucinous adenocarcinoma, prognosis, overall survival

## Abstract

*Background and Objectives:* Mucinous adenocarcinoma (MAC) and mucinous components (MCP) in colorectal cancers (CRC) have shown conflicting results regarding their prognostic impact. This study aims to evaluate survival differences between MAC, MCP, and non-mucinous adenocarcinoma (nMAC) in stage II and III CRC patients. *Materials and Methods:* 224 CRC patients who underwent surgery between 2013 and 2021 were analyzed retrospectively. Patients were classified as nMAC, MCP, or MAC based on the percentage of extracellular mucin. Those who received neoadjuvant therapy, had stage I or IV TNM disease, and emergency cases were excluded. Survival analysis was performed using Kaplan–Meier curves and Cox regression models. *Results:* MAC patients showed worse survival outcomes compared to nMAC (*p* = 0.025). No difference in survival was found between MCP and nMAC (*p* = 0.055). Multivariate analysis identified MAC (OR: 2.814; *p* = 0.014) and perineural invasion (PNI) (OR: 2.283; *p* = 0.008) as independent factors associated with worse survival. Kaplan–Meier analysis revealed MAC’s worse prognosis than nMAC (*p* = 0.027). *Conclusions:* MAC was shown to have a worse prognosis than nMAC in stage II and III CRC patients, while MCP survival rates were similar with nMAC. These findings suggest that MAC requires more careful treatment approaches, while MCP and nMAC have better survival rates. Further studies focusing on molecular and genetic profiles are needed to better understand these outcomes.

## 1. Introduction

Patient-specific treatment modalities are gaining importance in cancer patients with each passing day [1]. Factors such as the localization of lesions, histological subtypes, and immunohistochemical markers are associated with this topic in colorectal cancers [2,3]. Many studies have been conducted, particularly on the stage of colon cancer and the integrity of the resected specimen, and research continues in this area.

The most common histological subtype of colorectal cancers (CRCs) is adenocarcinoma. Mucinous CRC, on the other hand, is a rarer subtype. Unlike conventional adenocarcinomas, mucinous CRC is characterized by mucin components making up more than half of the tumor volume. While mucinous CRC accounts for approximately 20% of all CRC adenocarcinomas, its prevalence is higher in Western countries and lower in Eastern countries [4].

Mucinous colorectal cancers (MAC) are reported to originate more frequently from the proximal colon compared to other colorectal adenocarcinomas when histopathological characteristics are evaluated. They also exhibit a higher tendency for lymph node infiltration and peritoneal metastasis [4,5]. Beyond macroscopic features, genetic predispositions play a role in MACs, with mutations in the B-RAF proto-oncogene (BRAF) and v-Ki-ras2 (KRAS) being commonly observed. Both mutations lead to the activation of the RAS/MAPK pathway, which results in increased cell proliferation and suppressed apoptosis [6]. Additionally, it has been reported that 70% of MACs are associated with microsatellite instability-high (MSI-H) and chromosomal instability [7]. Due to this genetic profile, these patients may exhibit a better response to immunotherapy compared to other subtypes and may have a more favorable prognosis. However, survival studies on MAC present conflicting findings regarding prognosis, leading to an ongoing debate.

When evaluating the genetic alterations in pathology related to mucin production, it is noted that mucin, which normally serves a protective secretory function beneath the epithelium, exhibits different subtypes depending on its location throughout the gastrointestinal system. In the human mucin family, secreted mucins have organ-specific characteristics. During oncogenic activation, a decrease in or complete loss of mucus expression specific to the affected region can be observed. Among mucin subtypes, Mucin-2 (MUC2), predominantly produced in the proximal colon, and MUC5AC, primarily expressed in gastric and tracheobronchial epithelium, have been shown to play a role in the development of MAC. These characteristics have led to the exploration of alternative treatment approaches for MAC patients. In adenocarcinoma cases that contain mucin but have a mucin density of less than 50%, although similarities in mutation types have been reported, their prognostic trends have been shown to differ from those of MAC [8,9].

In studies investigating the relationship between mucinous histology (MH) subtypes in colorectal adenocarcinomas and prognosis, no consistent conclusion has been reached. When comparing colorectal carcinomas with at least 5% mucinous component to those without, it has been shown that there is no independent factor affecting prognosis between the groups [10]. In another retrospective analysis, it was demonstrated that patients with MAC have similar survival rates to those with mucinous component (MCP) or non-mucinous adenocarcinoma (nMAC) CRC. Additionally, it has been reported that when the mucin content exceeds 70%, more poor prognostic indicators are identified, and survival is negatively affected [11]. However, in contrast, a recent systematic review and meta-analysis found that CRC patients with signet-ring cell (SC) or MAC are associated with later-stage diagnosis, right-sided colon origin, higher recurrence rates, and worse prognosis compared to other histological subtypes [12].

When colon and rectal cancers are evaluated separately, different outcomes are reported in the literature. In stage 3 colon cancers, mucinous adenocarcinomas have been shown to have a worse prognosis compared to other histological types, while this effect was noted to be insignificant when compared with stage 2 cases [13]. In rectal cancers, studies have also demonstrated that the presence of MAC is associated with poor prognosis [14].

In this patient group where there is still no clear consensus in the literature, genetic phenotyping and microbiome analyses are ongoing to explain this situation. These studies mainly focus on comparing MAC and SC histological subtypes, while studies that specifically compare MAC and MCP with nMAC are limited in the literature. This study aims to evaluate whether the presence of MAC or MCP leads to a difference in survival compared to other colorectal cancers.

## 2. Materials and Methods

### 2.1. Study Design and Ethical Approval

This retrospective cohort study examines the records of CRC patients who underwent surgery at the Gastroenterologic Surgery Department of University of Health Sciences Kosuyolu Training and Research Hospital. The study covered the period between 1 January 2013, and 31 December 2021. Ethical approval for the study was obtained before data collection, with decision number 2024/05/794 from the Ethics Committee of the same institution on 5 March 2024. The study followed the principles of the Declaration of Helsinki and relevant ethical guidelines, ensuring that patient confidentiality and privacy were strictly maintained throughout the research.

### 2.2. Inclusion Criteria

Patients were selected for the study according to specific criteria to maintain consistency and align with the research goals. The inclusion criteria were as follows: patients who underwent surgery for histologically confirmed TNM Stage 2 or Stage 3 CRC with a curative surgical intent; those who underwent right hemicolectomy for cecum, ascending colon, hepatic flexure, and transverse colon tumors, left hemicolectomy for left colon tumors, or anterior resection for sigmoid colon tumors in accordance with the principles of complete mesocolic excision; those who underwent low anterior resection or abdominoperineal resection for rectal tumors in accordance with the total mesorectal excision principles; patients older than 18 years; and those with complete clinicopathological features and follow-up data.

### 2.3. Exclusion Criteria

To maintain the homogeneity and specificity of the study, the following exclusion criteria were applied: patients who underwent palliative or emergency surgery, and those who did not have an R0 resection. Cases with inadequate dissection criteria (positive surgical margins, surgical specimens with fewer than 12 lymph nodes or R1-2 resections) were excluded to focus on complete and oncologically appropriate surgical procedures. Additional exclusion criteria included patients not operated on according to oncological principles, patients who were TNM stage I or IV, and those with postoperative follow-up durations of less than 30 days. Lastly, to avoid bias in survival analysis, colon and rectal cancer patients who had received neoadjuvant therapy were excluded from the study.

### 2.4. Data Collection

Demographic information, preoperative tumor markers, previous operation records, history of neoadjuvant therapy, pathology data, operation durations, postoperative complications during follow-up, length of hospital stay (LOS), and survival data were retrospectively reviewed for all patients. American Society of Anesthesiology (ASA) scores and Body Mass Index (BMI) were recorded based on previous studies [15,16]. Preoperative histories and, if available, treatment data were extracted from patient record systems. Postoperative follow-up data were also collected from the patient record system when available; if unavailable, data were retrieved through the registered contact details. Only cancer-related death patients were included. Variables related to pathology results and staging were classified according to the eighth edition of the Union for International Cancer Control-American Joint Committee on Cancer (UICC-AJCC) TNM Classification System [17]. Patients’ CRC histological types were divided into three groups based on their MH: in accordance with World Health Organization (WHO) criteria for the definition of mucinous components, tumors in which extracellular mucin constitutes less than 5% of the tumor volume, were classified as non-mucinous adenocarcinoma (nMAC); those with 5–50% mucin content were categorized as mucinous carcinoma with partial mucinous component (MCP); and those with more than 50% mucin content were classified as mucinous adenocarcinoma (MAC) [18]. Lastly, postoperative complications were evaluated and grouped as minor and major based on the Clavien–Dindo Classification [19,20].

### 2.5. Surgery and Followup

**Figure 1:**
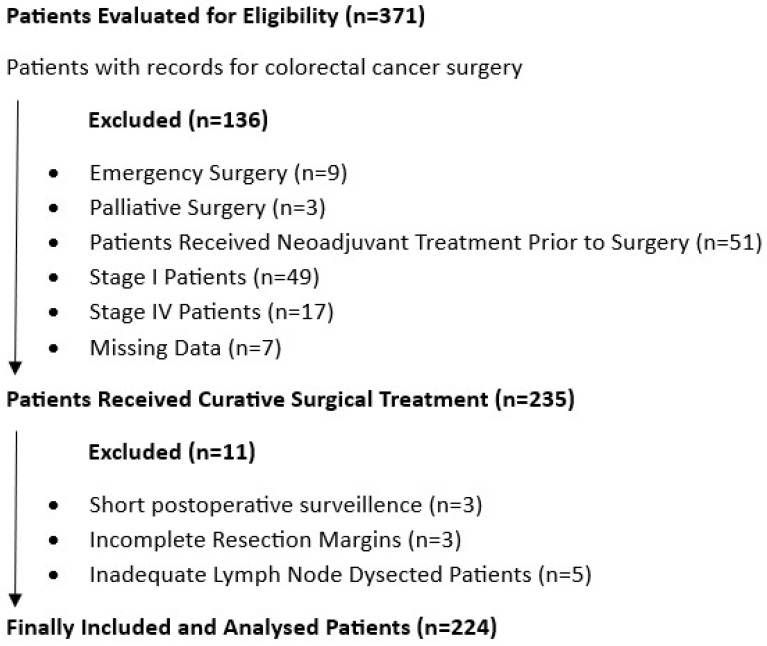
Flowchart of the study design and patient data enrolment.

Data was collected from 371 individuals who underwent surgery at our center between 2011 and 2021. Of this group, 136 were excluded for various reasons, including emergency surgery (9 cases), palliative surgery (3 cases), a history of neoadjuvant treatment (51 cases), TNM Stage I patients (49 cases), TNM Stage IV patients (17 cases), and missing data (7 cases). Additionally, three subjects were excluded due to a postoperative follow-up period of less than 3 months. Another eight patients were excluded due to inadequate dissection in pathology reports, including three patients with positive resection margins and five patients with an inadequate lymph node count. The mortality data of the patients were accessed from the national database, and only tumor-related deaths were included. For those with recorded death dates, the duration from the surgery date to the date of death was calculated to determine overall survival. Finally, 224 participants met the inclusion criteria (Figure 1).

### 2.6. Statistical Analysis

The software IBM^®^ SPSS^®^ (Statistical Package for the Social Sciences) version 25 (IBM Corp., Armonk, NY, USA) was used for statistical analysis. The distribution of numerical data was assessed using the Kolmogorov–Smirnov test. A normal distribution was found for survival groups, where comparisons were made using Student’s *t*-test, while a normal distribution was observed for MH subtypes, which were compared using the one-way ANOVA test. Qualitative data were presented as frequency and percentage. Continuous data were shown as mean and standard deviation (SD) for parametric values. Cox regression analysis was performed to evaluate factors influencing survival. Survival analysis formed by using the Kaplan–Meier method, with a significance level of 0.05 applied to all tests.

## 3. Results

A total of 224 patients were included in the study. According to survival data, 168 patients were in the “alive” group, while 56 were in the “not-alive” group. There were no T1 tumors in either group. The distribution of T2 tumors was similar between the groups (3% vs. 3.6%). However, T3 tumors were more frequent in the alive group (86.9% vs. 67.9%), whereas T4 tumors were more common in the not-alive group (10.1% vs. 28.6%) (*p* = 0.003). When assessing the presence of perineural invasion (PNI), it was found to be higher in the not-alive group (22.6% vs. 41.1%; *p* = 0.007). Regarding MH subtypes, nMAC was more frequent in the alive group (76.8% vs. 58.9%), MCP was similarly distributed between both groups (11.3% vs. 16.1%), and MAC was more prevalent in the not-alive group (11.9% vs. 25.0%) (*p* = 0.025). Patients in the not-alive group were older (62.02 ± 11.88 vs. 64.46 ± 13.59; *p* = 0.015) and had longer hospital stays (10.44 ± 7.08 vs. 13.63 ± 12.77; *p* = 0.020). There were not differences for Gender, Tumor location, N stage, Lymphovascular Invasion [LVI], Grade, TNM Stage, Complications, BMI, ASA scores or anastomosis leak rates in the analysis between the survival groups (*p* > 0.005) (Table 1).

When evaluated according to MH subtypes, differences were found in overall survival. While 20.3% of nMAC patients were in the not-alive group, this percentage was 32.1% for MCP patients and 41% for MAC patients (*p* = 0.025). All other variables including demographic (Gender, BMI, ASA Score and Age) and clinicopathologic (Tumor Site, T Stage, N Stage, LVI, PNI, Grade, TNM Stage, Complications, Anastomosis leak and LOS) showed similar distributions among the histologic groups (*p* > 0.005) (Table 2).

**Figure 2:**
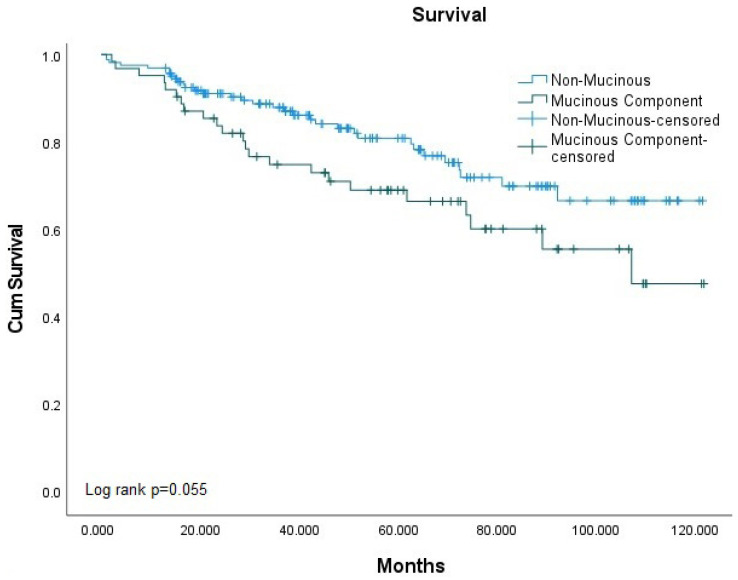
Overall Survival Analysis of Colorectal Cancer Cases Depending on Mucinous Component Presence.

**Figure 3:**
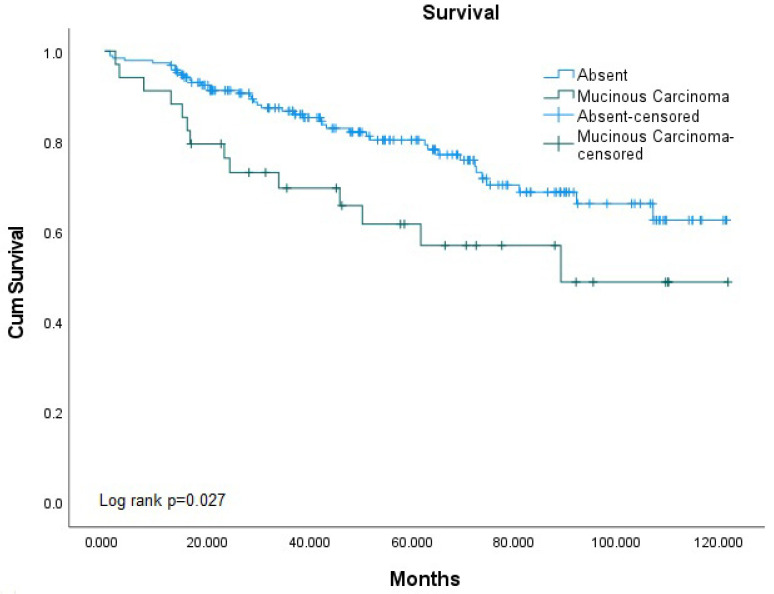
Overall Survival Analysis of Colorectal Cancer Cases Depending on Mucinous Carcinoma Presence.

In the survival analysis using the Kaplan–Meier test, survival rates were found to be similar between nMAC and MCP patients, although it was borderline (*p* = 0.055). However, when comparing nMAC and MAC patients, MAC patients showing worse survival results (*p* = 0.027) (Figure 2 and Figure 3).

All variables associated with survival in Table 1 were subjected to univariate regression analysis. While the T stage as a whole was found to be significant (*p* = 0.005), neither T3 tumors (Odds Ratio [OR]: 0.365, *p* = 0.610) nor T4 tumors (OR: 2.353, *p* = 0.345) demonstrated a statistically significant difference compared to T2 tumors. The presence of perineural invasion (PNI) was associated with a poorer prognosis (OR: 2.384, *p* = 0.008). Additionally, mucinous histology (MH) was observed to be generally significant for survival (*p* = 0.029). Although the presence of MCP appeared to influence survival, survival rates remained similar (OR: 1.852, *p* = 0.170). In contrast, patients with MAC exhibited worse survival outcomes compared to those with nMAC (OR: 2.74, *p* = 0.012) (Table 3).

Since all parameters were found to be significant, these variables were included in the multivariate Cox regression analysis to evaluate their prognostic relation with mortality. When evaluated alongside other factors (PNI and mucinous histology), the T stage remained a significant predictor of survival (*p* = 0.031). However, none of its subcategories (T3, OR: 0.612, *p* = 0.580) (T4, OR:1.808, *p* = 0.532) were identified as independent risk factors for survival. The presence of PNI was also identified as an independent risk factor for survival (OR: 2.283, *p* = 0.020). Finally, when mucinous histology (MH) was evaluated alongside T stage and PNI, it was identified as an independent risk factor for mortality (*p* = 0.030). While the presence of MCP did not significantly impact prognosis (OR: 2.037, *p* = 0.133), the presence of MAC was found to be independently associated with increased mortality (OR: 2.814, *p* = 0.014) (Table 3).

## 4. Discussion

In this study, it was shown that the presence of MAC negatively impacts prognosis in colorectal cancers. No survival difference was found between nMAC and MCP. Based on these results, it should be considered that stage 2 and 3 MAC patients may have a shorter survival time. Therefore, the adjuvant treatment and follow-up processes for these patients should be handled differently compared to nMAC patients, taking into account other prognostic markers.

There are many variables that play a role in determining the prognosis of colorectal cancers. Certain factors in histopathological examinations can also influence prognosis. Therefore, not every patient is evaluated in the same way, and even among patients at the same stage, survival expectations may differ. Basic factors such as stage, tumor perforation and the quality of the surgical specimen are crucial, but more specific analyses, including tumor microenvironment, microbiome studies, and genetic profile differences, have also been observed to cause changes in survival outcomes [21,22].

Mucinous histology remains a histological variable without a clear consensus. Generally, MAC forms 10–13% of all CRCs in European and U.S. data. While the frequency increases in patients over 65 years old in U.S. data, Chinese studies report a higher prevalence under the age of 50 [23]. Pathophysiologically, excess mucin production in MAC is linked to chemoresistance, along with mutations in KRAS, BRAF, APC, and p53, contributing to delayed apoptosis [24,25]. The presence of MAC or MCP has also been reported to negatively affect pathological complete response, with worse outcomes in terms of tumor downstaging and overall survival rates even in different neoadjuvant treatment strategies in rectum cancers [14]. Despite these findings, survival analyses remain inconclusive.

When considering mucin secretion in conjunction with genetic factors, MUC2 and MUC5AC expressions have been identified as the mucin markers most strongly associated with mucinous histology (MH). In non-mucinous adenocarcinomas (nMACs), suppression of these markers has been linked to mucosal inflammation, which in turn promotes epithelial proliferation and contributes to neoplastic transformations. Conversely, MUC2 overexpression has been recognized as a hallmark of mucinous adenocarcinoma (MAC). However, when comparing the pathophysiology of MAC to that of nMACs, several tumor-promoting mechanisms have been proposed. These include the formation of a mucin barrier surrounding tumor cells, which serves as a physical shield against therapeutic agents and immune responses, the suppression of apoptotic signaling pathways, p53 downregulation, and the inhibition of ligand expression necessary for immune cell activation in the tumor microenvironment. This interplay highlights a paradoxical balance in the oncologic outcomes of mucinous histology, where mucin production may simultaneously promote tumor progression while influencing its response to therapy [26].

In a study from 2019, 224 CRC patients with MCP were compared to 499 stage-matched nMAC patients. MCP was defined as having more than 5% extracellular mucin, which also included MAC cases. The analysis found similar survival rates between both groups. It was shown that, apart from tumor stage, no other variable had a significant effect on survival. Even when comparing only MAC cases, there was no survival difference. In univariate analysis, SC and MSI-High appeared significant, but multivariate analysis showed they did not affect prognosis. However, most of the patients in that study were stage 3 and 4, with 20% of nMAC and 35% of MCP cases being stage 4. This might limit the ability to see the impact of MH or other prognostic markers. In our study, only stage 2 and 3 patients were included. Like the mentioned study, patients who received neoadjuvant therapy were excluded to ensure homogeneity. In our study, although borderline, similar survival rates were found between MCP and nMAC cases. However, significant survival differences were seen between MAC and nMAC cases [26].

In a similar study, 112 patients with MAC were matched with 336 patients with nMAC and compared. Only patients who received postoperative adjuvant therapy were included in the study. MAC patients were compared with nMAC and MRC patients. In their analysis, both overall survival and disease-free survival were shown to be worse in the MAC group and in Stage III patients. However, no difference in overall or disease-free survival was observed between MAC and nMAC cases in Stage II patients. In our study, only Stage II and III patients were included, and similar survival rates were found in both stages. However, overall survival was found to be lower in the presence of MAC. In our study, patients with nMAC and those with only MCP were evaluated separately, and it was found that survival rates were similar between these groups [13].

In another study, MAC and nMAC cases were compared. By including patients with Stage I and Stage IV, a total of 372 patients were analyzed. The presence of MAC was found to be associated with more T4 tumors, poor differentiation, and extranodal invasion. Additionally, there was a significant decrease in both overall and disease-free survival times in the presence of MAC. However, in multivariate analyses, MAC was not found to have an effect on survival. In our study, the presence of MAC was shown to be an independent factor affecting survival. We believe that the reason for this difference is the exclusion of all stages, particularly metastatic cases, from our study [27].

In the most relevant study to our research, patients were classified according to the presence of MAC, nMAC, and MCP based on their pathology reports. The study concluded that the presence of MAC did not influence survival outcomes. However, in the ROC analysis conducted only on stage 2–3 patients, it was reported that survival was affected when the mucinous component percentage was over 70%. Tumor localization in this study was divided only into right or left colon, and rectal cancers, as well as patients who received neoadjuvant therapy, were not specified. In contrast, our study demonstrated that the presence of MAC negatively impacted survival, regardless of stage. However, this effect was not observed in cases where MCP was present [11].

### 4.1. Future Directions

In our study, when analyzing the pathological data from the retrospective dataset, variables such as age, T stage, and PNI—previously identified in the literature as associated with survival—did not show significant differences in our cohort. Additionally, other factors reported in previous studies, including tumor grade, N stage, TNM stage, and LVI, exhibited a similar distribution across the three-group analysis.

Previous studies on clinicopathological characteristics of patients with mucinous histology (MH) have found no significant differences, except for a higher frequency of proximal tumor localization and lymph node involvement. Given this, it is expected that genetic subtyping studies will provide more comprehensive insights into both tumor biology and prognosis in patients with MH. Future large-scale cohort studies assessing mucin production, particularly MUC2 and MUC5AC expression, alongside genetic mutations associated with CRC development, could provide a clearer understanding of the relationships between nMAC, MCP, and MAC [4].

Furthermore, since MACs are more frequently observed in the proximal colon and demonstrate a higher response to immunotherapy, prospective studies investigating the underlying mechanisms of this association could yield valuable insights. Beyond well-known genomic markers such as MSI, BRAF, and KRAS, evaluating molecular markers specific to the proximal colon would be beneficial [24].

Notably, Farnesoid X receptors (FXR)—which are predominantly found in the mucosa of the proximal colon and terminal ileum and play a crucial role in bile acid absorption and immune modulation—have been implicated in the pathophysiological mechanisms of CRC. FXR upregulation or downregulation exhibits a paradoxical proto-oncogenic effect similar to the increase in or suppression of mucin density. It has been shown that when MUC2 is downregulated and MUC1 is overexpressed, the mucin layer diminishes, leading to chronic inflammation and inhibition of apoptotic mechanisms. Similarly, excessive FXR activation has been linked to reduced mucin secretion, contributing to inflammatory bowel diseases and inflammation-associated neoplasms. A paradoxical pathology similar to mucinous histology is also observed in cases where FXR downregulation leads to decreased immune cell response and suppressed apoptosis which results in an aggressive behavior in CRC [28]. The immunomodulating effects of FXR have also been presented correlation with immunotherapy response in lung and breast cancers [29,30]. Given that both conditions are more commonly associated with CRC tumors located in proximally located, and similar immunotherapy response properties, future studies could incorporate FXR regulation and MH assessment alongside established genetic risk factors to further elucidate these mechanisms.

### 4.2. Strengths and Limitations

Our study has several limitations. Firstly, it was designed retrospectively. Although we had complete access to overall survival data, due to patients receiving adjuvant therapy at different centers, we were unable to obtain complete data on DFS, mutation analysis records, and adjuvant treatment. Additionally, compared to other large-scale analyses in the literature, our study has a smaller sample size. This situation may have led to the similar distribution of clinicopathologic factors, such as grade and lymph node involvement, which could affect survival among the groups. The additional molecular mutations in patients, which contribute to aggressive tumor behavior, could not be fully identified in the reports. Therefore, this variable could not be included. A prospective study focusing in comparison of KRAS, BRAF, APC, and p53 between the groups could provide more valuable insights into this phenomenon. However, by excluding Stage I and Stage IV patients, cases receiving neoadjuvant therapy, and patients with a history of emergency or palliative surgery potential bias was minimized, and a more isolated prognosis assessment was achieved.

In conclusion, our study found that MACs in Stage II-III colorectal cancers have a worse prognosis compared to nMACs. However, this poor prognostic effect was not observed in the presence of MCP. Larger-scale studies including only MAC or MCP patients, with comprehensive prognostic factor assessments and molecular analyses, are needed for further clarification of this conflicting issue.

## 5. Conclusions

In CRC patients, the presence of MAC negatively impacts prognosis, whereas the overall survival of MCP patients follow a similar course to those of nMAC patients. Based on postoperative pathology findings, different treatment strategies should be planned according to MAC and MCP subtypes. It is anticipated that larger-scale studies incorporating prognostic variables affecting disease-free survival and recurrence, as well as evaluating mucinous component data alongside genetic mutations, will contribute to the development of more patient-specific treatment modalities in the future.

## Figures and Tables

**Table 1:** Demographic and Pathologic Variable Analysis Depending on Overall Survival.

Variables		Alive	Not Alive	*p*, †
		n = 168 (%75)	n = 56 (%25)	
Gender	Male	107 (63.7%)	30 (53.6%)	0.178
	Female	61 (36.3%)	26 (46.4%)	
Tumor Site	Caecum	25 (14.9%)	8 (14.3%)	0.709
	Right Colon	40 (23.8%)	15 (26.8%)	
	Transverse Colon	8 (4.8%)	3 (5.4%)	
	Left Colon	13 (7.7%)	8 (14.3%)	
	Sigmoid Colon	55 (32.7%)	15 (26.8%)	
	Rectum	27 (16.1%)	7 (12.5%)	
T Stage	T2	5 (3.0%)	2 (3.6%)	0.003 **
	T3	146 (86.9%)	38 (67.9%)	
	T4	17 (10.1%)	16 (28.6%)	
N Stage	N0	112 (66.7%)	33 (58.9%)	0.165
	N1	37 (22.0%)	11 (19.6%)	
	N2	19 (11.3%)	12 (21.4%)	
LVI	Negative	113 (67.3%)	32 (57.1%)	0.170
	Positive	55 (32.7%)	24 (42.9%)	
PNI	Negative	130 (77.4%)	33 (58.9%)	0.007 **
	Positive	38 (22.6%)	23 (41.1%)	
Grade	Good	24 (14.3%)	6 (10.7%)	0.089
	Moderate	129 (76.8%)	39 (69.6%)	
	Poor	15 (8.9%)	11 (19.6%)	
TNM Stage	II	114 (67.9%)	33 (58.9%)	0.223
	III	54 (32.1%)	23 (41.1%)	
Complications	Minor	99 (59.0%)	33 (58.9%)	1.000
	Major	69 (41.0%)	23 (41.1%)	
BMI	Underweight	3 (1.8%)	0 (0.0%)	0.087
	Normal Weight	56 (33.3%)	16 (28.6%)	
	Overweight	78 (46.4%)	21 (37.5%)	
	Obesity	31 (18.5%)	19 (33.9%)	
ASA Score	1	2 (1.2%)	0 (0.0%)	0.113
	2	44 (26.2%)	17 (30.4%)	
	3	118 (70.2%)	34 (60.7%)	
	4	4 (2.4%)	5 (8.9%)	
Anastomosis Leakage	No	153 (91.1%)	49 (87.5%)	0.437
	Yes	15 (8.9%)	7 (12.5%)	
Mucinous Histology	Non-Mucinous	129 (76.8%)	33 (58.9%)	0.025 *
	Mucinous Component	19 (11.3%)	9 (16.1%)	
	Mucinous Carcinoma	20 (11.9%)	14 (25.0%)	
		Mean ± SD		*p* ‡
		Alive	Not Alive	
Age		62.02 ± 11.88	64.46 ± 13.59	0.015 *
LOS/Days		10.44 ± 7.08	13.63 ± 12.77	0.020 *

LVI: Lymphovascular Invasion, PNI: Perineural Invasion, BMI: Body Mass Index, ASA: American Society of Anesthesiology, LOS: Length of Hospital Stay, SD: Standard Deviation, * *p* < 0.05, ** *p* < 0.01, † Chi-Square, ‡ Independent t Test.

**Table 2:** Demographic and Pathologic Variable Analysis Depending on Mucinous Histology Subtypes.

Variables		nMAC (n = 162)	MCP (n = 28)	MAC (n = 34)		*p*, †
Gender	Male	103 (63.6%)	17 (60.7%)	17 (50.0%)		0.335
	Female	59 (36.4%)	11 (39.3%)	17 (50.0%)		
Tumor Site	Caecum	21 (13.0%)	3 (10.7%)	9 (26.5%)		0.054
	Right Colon	33 (20.4%)	10 (35.7%)	12 (35.3%)		
	Transverse Colon	8 (4.9%)	3 (10.7%)	0 (0.0%)		
	Left Colon	14 (8.6%)	4 (14.3%)	3 (8.8%)		
	Sigmoid Colon	58 (35.8%)	5 (17.9%)	7 (20.6%)		
	Rectum	28 (17.3%)	3 (10.7%)	3 (8.8%)		
T Stage	T2	4 (2.5%)	1 (3.6%)	2 (5.9%)		0.666
	T3	136 (84.0%)	23 (82.1%)	25 (73.5%)		
	T4	22 (13.6%)	4 (14.3%)	7 (20.6%)		
N Stage	N0	108 (66.7%)	17 (60.7%)	20 (58.8%)		0.667
	N1	34 (21.0%)	5 (17.9%)	9 (26.5%)		
	N2	34 (21.0%)	6 (21.4%)	5 (14.7%)		
LVI	Negative	105 (64.8%)	16 (57.1%)	24 (70.6%)		0.544
	Positive	57 (35.2%)	12 (42.9%)	10 (29.4%)		
PNI	Negative	115 (71.0%)	22 (78.6%)	26 (76.5%)		0.616
	Positive	47 (29.0%)	6 (21.4%)	8 (23.5%)		
Grade	Good	22 (13.6%)	3 (10.7%)	5 (14.7%)		0.151
	Moderate	126 (77.8%)	21 (75.0%)	21 (61.8%)		
	Poor	14 (8.6%)	4 (14.3%)	8 (23.5%)		
TNM Stage	II	108 (66.7%)	17 (60.7%)	20 (58.8%)		0.612
	III	54 (33.3%)	11 (39.3%)	14 (41.2%)		
Complications	Minor	93 (57.4%)	18 (64.3%)	21 (61.8%)		0.741
	Major	69 (42.6%)	10 (35.7%)	13 (38.2%)		
BMI	Underweight	3 (1.9%)	0 (0.0%)	0 (0.0%)		0.555
	Normal Weight	55 (34.0%)	10 (35.7%)	7 (20.6%)		
	Overweight	71 (43.8%)	10 (35.7%)	18 (52.9%)		
	Obesity	33 (20.4%)	8 (28.6%)	9 (26.5%)		
ASA Score	1	2 (1.2%)	0 (0.0%)	0 (0.0%)		0.208
	2	39 (24.1%)	13 (46.4%)	9 (26.5%)		
	3	113 (69.8%)	14 (50.0%)	25 (73.5%)		
	4	8 (4.9%)	1 (3.6%)	0 (0.0%)		
Anastomosis Leak	Negative	145 (89.5%)	27 (96.4%)	30 (88.2%)		0.481
	Positive	17 (10.5%)	1 (3.6%)	4 (11.8%)		
Overall Survival	Alive	129 (79.6%)	19 (67.9%)	20 (58.8%)		0.025 *
	Not Alive	33 (20.4%)	9 (32.1%)	14 (41.2%)		
		Mean ± SD			F	*p*, §
Age		62.94 ± 12.00	58.68 ± 13.04	64.44 ± 13.07	1.865	0.157
LOS		11.17 ± 7.65	9.79 ± 4.79	12.74 ± 15.15	0.853	0.428

nMAC: Non-mucinous Cancer, MCP: Mucinous Component, MAC: Mucinous Adenocarcinoma, LVI: Lymphovascular Invasion, PNI: Perineural Invasion, BMI: Body Mass Index, ASA: American Society of Anesthesiology, LOS: Length of Hospital Stay, SD: Standard Deviatio, * *p* < 0.05, † Chi-Square, F: F-statistic used in ANOVA test, § One-way ANOVA test.

**Table 3:** Cox Regression Analysis for Dependents Affecting Survival.

			Univariate			Multivariate	
Prognostic Factors		OR	95% CI	*p*	OR	95% CI	*p*
	T2			0.005 **			0.031 *
T Stage	T3	0.651	0.121–3.485	0.616	0.612	0.108–3.474	0.580
	T4	2.353	0.398–13.900	0.345	1.808	0.282–11.586	0.532
PNI		2.384	1.253–4.538	0.008 **	2.283	1.140–4.572	0.020 *
	nMAC			0.029 *			0.030 *
MH	MCP	1.852	0.768–4.466	0.170	2.037	0.805–5.152	0.133
	MAC	2.736	1.251–5.986	0.012 *	2.814	1.233–6.418	0.014 *

PNI: Perineural Invasion, MH: Mucinous Histology, nMAC: Non-mucinous Cancer, MCP: Mucinous Component, MAC: Mucinous Adenocarcinoma, OR: Odds Ratio, CI: Confidence Interval, * *p* < 0.05, ** *p* < 0.01.

## Data Availability

The datasets generated and analyzed during the current study are available from the corresponding author upon reasonable request.
